# Supplementary material for: Learning curve and surgical outcome of robotic assisted colorectal surgery with ERAS program
Source: Sci Rep. 2022 Nov 29;12:20566. doi: 10.1038/s41598-022-24665-w (PMC9709162; doi:10.1038/s41598-022-24665-w)
Supplement: Supplementary file 1 — Supplementary Information 1. [file 41598_2022_24665_MOESM1_ESM.zip › LSC Raw data-Ñ[▒K/075/O_ñw╝╨░O▒Kñσ.pdf]

正式摘要(電子簽章：0000001880288881)

請選擇...reporter

臺中榮民總醫院

Operation Note

[redacted]

- 手術日期：2020/03/26
- 手術醫師：VS 蔣鋒帆，CR 黃俋霖，R 陳映菁
- Anesthesia : GE
- Position : Lithotomy
- Aseptic agents : Betadine Alcohol

Diagnosis

- Pre-operative diagnosis : Malignant tumor of rectum
- Post-operative diagnosis : Ditto.

Surgical Indication

- For oncologic resection

Operation

- Low anterior resection with colo-anal anastomosis+ Ileostomy+ Laparoscopy

Finding

- Operative findings:
  1. A 4x5cm Bormann's type III ulcerative tumor at low rectum, which invaded to the muscular layer but didn't penetrate through the serosa.
  2. There was no visible liver metastasis, no visible peritoneal seeding.
  3. Some adhesion between sigmoid colon and abdomen wall

  Operative methods:
  1. Laparoscopic low anterior resection with end-to-end anastomosis, EEA 29mm. air leak test: done.
  2. Loop ileostomy at RLQ of abdomen.
  3. High ligation (+) IMA above the left colic artery.
  4. Cut margin: proximal 18 cm, distal 1 cm.
  5. Take down of splenic flexure: nil.
  6. Re-peritonealization: nil.
  7. A 12mm penrose placed in the pelvis.
  8. 10x13cm Surgiwrap was placed

Procedure

- General endotracheal anesthesia was induced and then the patient was placed in modified Trendelenburg lithotomy position. Preoperative antibiotics were given. A Foley catheter and nasogastric tube were placed. The abdomen was prepped and draped in standard sterile fashion. A small vertical incision was made at low midline abdomen and a 12mm trochar was inserted into the peritoneal cavity by open method. Peumoperitoneum was established with CO2 inflation. Laparoscopy was inserted and then two 5mm trochars were inserted over RLQ and low midline. Laparoscopy was inserted via a 5mm trochar site. The operative findings were mentioned as above. The small bowel was inspected and retracted to the right by grasper. The sigmoid colon was then held by grasper and the peritoneum overlying the mesentery was then scored with electrocautery. The inferior mesentery artery was identified, ligated near its origin from the aorta, and transected, so was inferior mesentery vein. Dissection was done above the Gerota's fascia and toward the peritoneal attachments. The colon was mobilized from retroperitoneum overlying the sacral promontory and into the pelvis. The incision was extended laterally and medially across the peritoneal reflection. The mesorectum was then mobilized using sharp dissection. Distal descending

- colon and rectum were mobilized. A midline incision was made at paraumblical. The sigmoid colon was delivered into the incision. It was determined that a 29 mm circular stapler could be accommodated. A purse-string suture of 2-0 Prolene was placed, the anvil was inserted, and then the purse-string suture was tied. The rectal stump was irrigated with distilled water and then the circular stapler was introduced into the rectal stump. The proximal bowel with anvil in place was engaged with the circular stapler and the stapler was then fired. Two complete donuts were obtained and submitted to pathology. One 12mm penrose was placed at the pelvis and one 10x13cm SurgiWrap was placed between abdominal wall and peritoneal cavity.
  Then, we selected a loop of ileum at 20cm proximal to ileocecal valve.The ileum was
  pulled out of the abdomen and a colostomy rod was penetrated through the mesentary. The fascia was closed with a running suture of No.1 Vicryl. Subcutaneous tissues were closed with interrupted 3-0 Polysorb and the skin was approximated with Steri-Stripes. The ileostomy was matured with 3-0 chromic catgut suture with nipple at proximal limb. The patient tolerated the procedure well and was brought to the POR in stable condition.
- Estimated blood loss: minimum
- 器械數量: 正確
- 紗布數量: 正確

Image

- PAINT
  - colon & rectum

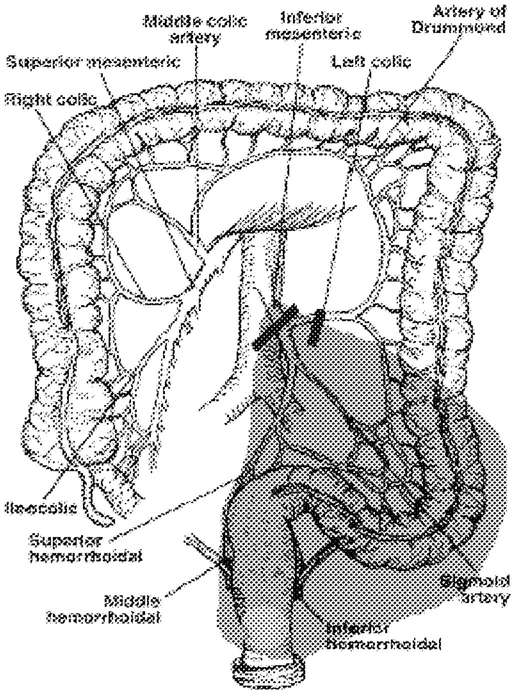

- 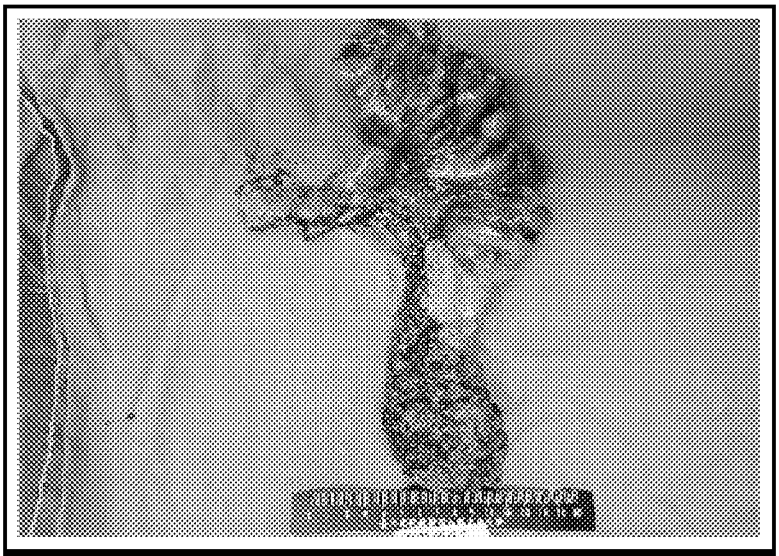
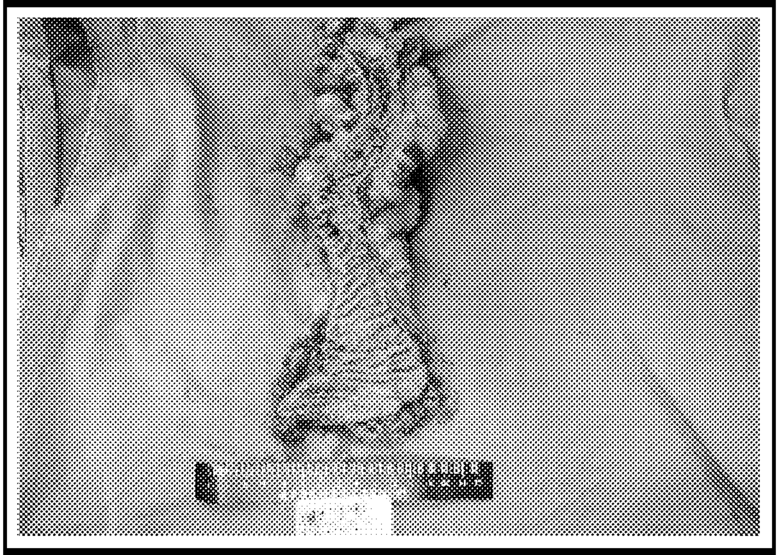
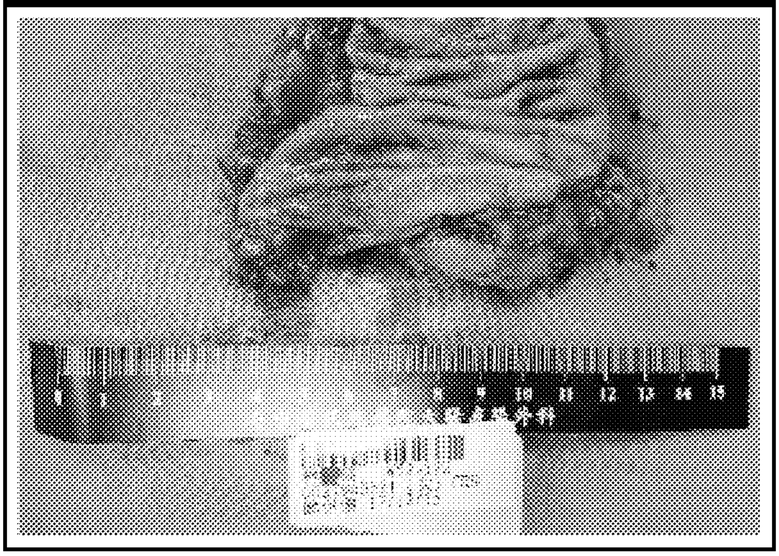

編輯主任 2062F蔣鋒帆、主治 2286H黃俋霖

正式摘要(電子簽章：000000188028881)

請選擇...reporter

doctorRank:B
